# Biobanking in LMIC settings for infectious diseases: Challenges and enablers^☆^

**DOI:** 10.1016/j.bsheal.2022.07.002

**Published:** 2022-10

**Authors:** Sameera Ezzat, Ruzica Biga, Zisis Kozlakidis

**Affiliations:** a National Liver Institute, Menoufia University, Shibin el Kom 32511, Egypt; b Mounir Armanious Research Center (MARC), Giza 12561, Egypt; c University Cote D’Azur, Nice 06103, France; d International Agency for Research on Cancer, World Health Organization, Lyon 69372, France

**Keywords:** Biobanking, Research infrastructures, Infectious diseases, Low-and-middle income countries, LMICs

## Abstract

Biobanking facilities are well established in high-income settings, where substantial funding has been invested in infrastructure. In contrast, such facilities are much less developed in resource-restricted settings. However, low-and middle-income countries (LMICs) still face a disproportionately high infectious diseases burden. Thus, the further development of infrastructure facilities, including biobanks is warranted as an important component of this unfolding clinical research environment. This perspective manuscript summarises the challenges and enablers for biobanking in LMICs, with a particular focus on infectious diseases, incorporating some of the lessons learned from the recent coronavirus disease 2019 (COVID-19) pandemic.

## Highlights

• Most biobanks in low-and middle-income countries (LMICs) have been set as part of specific project, thus resulting to a fragmented and siloed infrastructure landscape.
• Current biobanking challenges are represented by the following thematic groups: Equipment, Laboratory Management Information Systems (LIMS/BMIS), training and staff, ethical and legal frameworks.
• Biobanking enablers include, but are not limited to: the co-development of equipment, the interoperability of systems through data harmonization practices, the availability of LMIC-specific training material and courses.
• Local contexts and collaborations with existing infrastructures and /or surveillance networks in LMICs are likely to characterise the next period of biobanking.

## 1 Introduction

Biobanks are collections of biological material and associated data, stored in an organized system, where the samples and associated data can be provided for downstream research and/or clinical purposes and can even sometimes be linked to the person(s) who provided the sample [1]. Biobanks are increasingly recognised as foundational infrastructures that form the cornerstone of modern research, as they are able to provide systematically collected, standardised and research-ready biological material and associated data. This is particularly impactful as a number of research approaches are moving towards high-throughput settings, and as such biobanks are able to satisfy that need for increased volumes of standardised biological material and associated data [2].

Therefore, several institutions have embarked on generating large population cohorts that depend on large-scale biobanking facilities [3]. As the burden of disease is more severe in low-and middle-income countries (LMICs), as defined by The World Bank [4], there is a definite need in LMICs to replicate the strong international trend towards the development of such infrastructure and facilities, providing a means for LMICs inclusion in international research activities. This perspective manuscript summarises the challenges and enablers for biobanking in LMICs, with a particular focus on infectious diseases, incorporating some of the lessons learned from the recent coronavirus disease 2019 (COVID-19) pandemic.

## 2 Biobanking challenges

Most biobank facilities in LMICs have been traditionally set-up as part of a particular project targeted at major health issues affecting the populations of these countries. For example, such biobanks frequently involved large numbers of participants and were often created in human immunodeficiency virus (HIV) treatment facilities to monitor and control the spread of infection [5]. This has been replicated with a number of infectious diseases, e.g., Tuberculosis (TB), Zika, Ebola, and most recently with COVID-19 dedicated biobanks [6]. In order for such biobanks to continue to be developed and operate successfully, the following five challenges (equipment, LIMS, dedicated facilities, skilled staff, governance framework) need to be overcome as presented below.

### 2.1 Equipment

The availability of equipment might appear as a simple task of importing task-specific pieces of technology, however in the case of LMICs they can often be legacy systems from specific projects, and as such not necessarily interoperable. Additionally, environmental conditions in most LMICs affect the performance of electronic equipment and consumables. As such the equipment and consumables would need to be customised – ‘tropicalized’- for use in such settings, for example taking into account the often very wide variations in humidity in LMICs [7], [8].

### 2.2 LIMS/BIMS

Similarly, laboratory information management systems (LIMS) -or a subset of LIMS known as Biobank information management systems (BIMS) - often are a legacy system, partially suitable for biobanking purposes, and customization and subsequent training requirements could be costly. Thus LIMS/BIMS providers would need to support the likely need for open access software for the requirements of the LIMCs laboratories [9], such as BAOBAB LIMS that is open access software developed as part of the Bridging Biobanking and Biomedical Research across Europe and Africa (B3Africa) project [10]. Without appropriate software to drive quality assurance programmes, operational and data workflow standardization, the aim of interoperability between databases to support downstream research, becomes difficult and challenging.

### 2.3 Training and staff

In LMICs, training materials are frequently scarce, seldom updated specifically for the LMICs context. Furthermore, access to dedicated biobanking courses is commonly in high-income settings, as such attendance and costs are major obstacles. This is additional to the general lack of highly skilled laboratory staff [11]. Thus, assigning personnel to biobanking activities is a challenging task in conditions of limited availability of trained staff (and training opportunities) in health care services. Moreover, the need for educated and experienced biobankers is often underestimated. In general, research centres do not employ dedicated trained personnel, with corresponding training material for infectious diseases biobanking. Where present, these personnel significantly contribute to the development of biobanks. [11].

### 2.4 Ethical and legal frameworks

Finally, the last two decades have seen a slow but consistent development of legal and ethical frameworks for biobanking in LMICs in general, with infectious diseases biobanking being a constituent part of that effort [12], [13]. However, this aspect is only in its very first steps, and still requires not only the training of ethical boards and researchers on appropriate ethical and data governance of biobanking (e.g., in the different consent types, and current data protection requirements), but also a communication with the wider public so that strong bonds are formed based on trust [14].

## 3 Biobanking enablers

While the list of challenges may often seem daunting, there are certain actions that can be taken in order to facilitate biobanks overcoming the challenges mentioned above, and summarized in Table 1. For example, there are some manufacturers who are forming partnerships and investing in research exploring low-cost innovation (e.g., in diagnostics for COVID-19). In such cases biobanks can form partnerships in order to assist in the evaluation of such products in the field and enable their customization to resource-restricted settings. In terms of LIMS/BIMS, interoperability is a key aspect, and LIMS should support the standardised collection of data, harmonized to international guidelines (e.g., Minimum Information About Biobank Sharing (MIABIS 2.0)[15]). Adherence to such guidelines, as well as data quality indicators, would allow the comparability between individual collections, for the purpose of sharing within the institution, as well as for participation in international studies. Additionally, Open Access Software should continue to be investigated as (at a minimum) entry mechanisms for the biobanking of infectious diseases.

**Table 1:** The five priority aspects identified for infectious diseases biobanks in low-and middle-income countries (LMICs), with the related challenges and enablers.

**Priority**	**Challenges**	**Enablers**
Equipment	- Legacy equipment - Interoperability - Equipment maintenance	- Synergies with existing initiatives/infrastructures - ‘Tropicalized’ equipment
Laboratory Management Information Systems (LIMS/BIMS)	- Legacy systems - Consistent data input - Data quality	- Open access software - Staff training - Data quality indicators
Customised training materials	- Lack of core material - Cost of training	- Dedicated LIMC courses - Utilizing existing resources
Skilled laboratory staff	- Low staff numbers - High staff turnover - Competing priorities	- Synergies with existing initiatives/infrastructures - Staff incentives
Regulatory frameworks for samples and data collection/sharing	- Different frameworks for samples and data collection/sharing - Lack of appropriate legal/ethical frameworks	- Introduction of expert consensus where framework is absent - Highlighting gaps in legal/ethical frameworks

Regular quality assessment should be undertaken for biological samples and data, so as to validate local protocols (e.g., for biosafety), but also to set the basis for eventual accreditation of the facilities. Having said that, the key bottleneck remains the availability of skilled staff who know and understand the local health priorities and scientific possibilities, and can advise those who want to use samples for research. Incentivising skilled staff to remain and dedicate at least part of their work to biobanking would be a key component for long-term operational sustainability of such biobanking efforts. Regarding the training opportunities, a number of training courses are currently planned specific to biobanking in LMICs, e.g., in Egypt and Morocco, and it is hoped that they will eventually contribute to alleviating the skilled staff availability (Kozlakidis, personal communication). Moreover, there is the opportunity for training material and best practices from biobanking organizations, such as the International Society for Biological and Environmental Repositories (ISBER), to be used as a basis for the further development of LMIC-specific training material.

There is a rising need for development of frameworks that explicitly deals with research on infectious disease samples, especially in LMIC with no regulatory guidance or whose health research legislation or guidelines do not cover developments in the area of biobanking and data protection [12]. Having said that, there is an increased identification of potential gaps and understanding for the need to create LMIC-appropriate ethical frameworks for biobanking, so that particular, local ethical and cultural norms can be taken into consideration [15], [16], [17]. Doing so is likely to strengthen the overall trust that will exhibit towards biobanks in the longer-term. Although, each one of these enablers can play a role in promoting the growth of biobanking in LMICs, perhaps the ‘easiest’ single action would be to build capacity and inclusivity through cooperation [18].

Already in the field of cancer biobanking, there is an LMIC-dedicated network supported by the World Health Organization (WHO) and the National Institutes of Health, called the Biobanking and Cohort Building Network (BCNet; https://bcnet.iarc.fr/). Collaborations of LMICs, e.g., a ‘South-South’ collaboration, such as the Africa Taskforce for Coronavirus (AFTCOR) or the European Virus Archive (EVA), are often better placed to find solutions to their specific challenges and needs, as has been shown in the case of COVID-19 and the inclusion of many sites in research consortia [19]. Similarly, the announced BioHub creation by the WHO is anticipated to foster international collaboration in the sharing of infectious diseases agents [20]. Within local contexts such collaborations can include existing tuberculosis [21], HIV [22], influenza [23], and other surveillance activities. In this manner the biobanking of infectious diseases will add value to already existing systems, and would be more likely to secure sustained funding longer-term.

## 4 Conclusion

The ongoing scientific discovery and technological developments will be underpinned by the availability of available infrastructures, and biobanking is an important component of this unfolding clinical research environment. Biobanking facilities are well established in high-income settings, where substantial funding has been invested in infrastructure. In contrast, such facilities are much less developed in resource-restricted settings. However, LMICs still face a disproportionately high infectious diseases burden. Thus, the further development of infrastructure facilities, including biobanks is warranted. Biobanks are being developed in LMICs, and many participate in infectious diseases research as evidenced by their inclusion in COVID-19 international consortia, but not in sufficient numbers reflecting on the population and clinical needs. The current perspective provided a high-level summary of the five most common challenges faced by infectious diseases biobanks in LMICs (equipment, LIMS, dedicated facilities, skilled staff, governance framework) and identified enablers that may overcome those challenges. Importantly, the collaboration with existing infrastructures may be a practical approach in allowing biobanks to demonstrate their added value and thus influencing the appropriate level of funding, resources, and institutional and public support.

## Author contributions

**Sameera Ezzat:** Formal Analysis, Writing ‐ Original Draft. **Ruzica Biga:** Writing ‐ Review & Editing, Writing ‐ Original Draft. **Zisis Kozlakidis:** Conceptualization, Methodology, Supervision, Writing ‐ Review & Editing.

## Conflict of interest statement

The authors declare that there are no conflicts of interest. Where authors are identified as personnel of the International Agency for Research on Cancer/WHO, the authors alone are responsible for the views expressed in this article and they do not necessarily represent the decisions, policy or views of the International Agency for Research on Cancer/WHO.

## References

[1] Organisation for Economic Co-operation and Development (OECD), Best practice guidelines for biological resource centres. https://www.oecd.org/sti/emerging-tech/38777417.pdf, 2012 (accessed 30 May 2022).

[2] Pietiainen V. (2014). The high throughput biomedicine unit at the institute for molecular medicine Finland: high throughput screening meets precision medicine. Comb. Chem. High Throughput Screen..

[3] Hainaut P., Vozar B., Rinaldi S., Riboli E., Caboux E. (2011). The European prospective investigation into cancer and nutrition biobank. Methods Mol. Biol..

[4] The World Bank, World Bank Country and Lending Groups: Country classification. https://datahelpdesk.worldbank.org/knowledgebase/articles/906519-world-bank-country-and-lending-groups, 2022 (accessed 30 May 2022).

[5] Thio C.L., Seaberg E.C., Skolasky R., Phair J., Visscher B., Munoz A., Thomas D.L. (2002). HIV-1, hepatitis B virus, and risk of liver-related mortality in the multicenter cohort study (MACS). Lancet.

[6] Abayomi A., Saka B., Osibogun A., Wright O., Idris J., Ogunsola F. (2021). From Ebola to COVID-19: emergency preparedness and response plans and actions in Lagos, Nigeria. Global Health.

[7] Jacobs J., Hardy L., Semret M., Lunguya O., Phe T., Affolabi D., Yansouni C., Vandenberg O. (2019). Diagnostic bacteriology in district hospitals in sub-Saharan Africa: at the forefront of the containment of antimicrobial resistance. Front. Med..

[8] Orekan J., Barbé B., Oeng S., Ronat J.B., Letchford J., Jacobs J., Affolabi D., Hardy L. (2021). Culture media for clinical bacteriology in low-and middle-income countries: challenges, best practices for preparation and recommendations for improved access. Clin. Microbiol. Infect..

[9] Klingström T. (2017). Data integration and handling (Vol. 2017, No. 2017: 99)..

[10] Bendou H., Sizani L., Reid T., Swanepoel C., Ademuyiwa T., Merino-Martinez R., Meuller A., Abayomi A., Christoffels A. (2017). Baobab laboratory information management system: development of an open-source laboratory information management system for biobanking. Biopreserv. Biobank.

[11] Mendy M., Caboux E., Sylla B.S., Dillner J., Chinquee J., Wild C., BCNet survey participants. (2014). Infrastructure and facilities for human biobanking in low-and middle-income countries: A situation analysis. Pathobiology.

[12] Vodosin P., Jorgensen A.K., Mendy M., Kozlakidis Z., Caboux E., Zawati M.H. (2021). A Review of regulatory frameworks governing biobanking in the low- and middle-income member countries of BCNet. Biopreserv. Biobank..

[13] Fernando B., King M., Sumathipala A. (2019). Advancing good governance in data sharing and biobanking-international aspects. Wellcome Open Res..

[14] Singh S., Cadigan R.J., Moodley K. (2021). Challenges to biobanking in LMICs during COVID-19: time to reconceptualise research ethics guidance for pandemics and public health emergencies?. J. Med. Ethics.

[15] Merino-Martinez R., Norlin L., van Enckevort D., Anton G., Schuffenhauer S., Silander K., Mook L., Holub P. (2016). Toward global biobank integration by implementation of the minimum information about biobank data sharing (MIABIS 2.0 Core). Biopreserv. Biobank..

[16] Singh S., Moodley K. (2021). Stakeholder perspectives on the ethico-legal dimensions of biobanking in South Africa. BMC Med. Ethics.

[17] Alahmad G., Dierickx K. (2018). Ethics of research biobanks: Islamic perspectives. Biopreserv. Biobank..

[18] Fanning J.P., Murthy S., Obonyo N.G., Baillie J.K., Webb S., Dalton H.J., Fraser J.F. (2021). Global infectious disease research collaborations in crises: building capacity and inclusivity through cooperation. Global Health.

[19] Coutard B., Romette J.L., Miyauchi K., Charrel R., Prat C.M.A., EVA Zika Workgroup, EVA COVID-19 Workgroup (2020). The importance of biobanking for response to pandemics caused by emerging viruses: the european virus archive as an observatory of the global response to the Zika virus and COVID-19 crisis. Biopreserv Biobank.

[20] Nature Biotechnology (2021). Around the world in a month. Nat. Biotechnol..

[21] Podewils L.J., Bantubani N., Bristow C., Bronner L.E., Peters A., Pym A., Mametja L.D. (2015). Completeness and reliability of the Republic of South Africa National Tuberculosis (TB) Surveillance System. BMC Public Health.

[22] Boothe, M. HIV Surveillance among key populations in Mozambique: Opportunities for enhanced prevention, care and treatment, Diss. Ghent University. https://biblio.ugent.be/publication/8731293, 2021 (accessed 30 May 2022).

[23] Broor S., Campbell H., Hirve S., Hague S., Jackson S., Moen A., Nair H., Palekar R., Rajatonirina S., Smith P.G. (2020). Leveraging the Global Influenza Surveillance and Response System for global respiratory syncytial virus surveillance—opportunities and challenges. Influenza Other Respir. Viruses.

